# Anatomy of a Discovery: M1 and M2 Macrophages

**DOI:** 10.3389/fimmu.2015.00212

**Published:** 2015-05-05

**Authors:** Charles Dudley Mills

**Affiliations:** ^1^Biomedical Consultants, Marine on St. Croix, MN, USA

**Keywords:** macrophages, innate immunity, M1, M2, wound, cancer, Th1/Th2

## Abstract

M1 and M2 macrophage-type responses kill or repair *in vivo*. The unique ability of macrophages to make these polar opposite type of responses provides primary host protection and maintains tissue homeostasis throughout the animal kingdom. In humans and other higher animals, M1 and M2-type macrophage responses also initiate and direct T cells/adaptive immunity to provide additional protection such as Th1 (cytotoxic) or Th2 (antibody-mediated) type responses. Hence, macrophages were renamed M1 and M2 to indicate the central role of macrophages/innate immunity in immune systems. These findings indicate that the long held notion that adaptive immunity controls innate immunity was backward: a sea change in understanding how immune responses occur. The clinical impact of M1/kill and M2/repair responses is immense playing pivotal roles in curing (or causing) many diseases including infections, cancer, autoimmunity, and atherosclerosis. How M1/M2 came to be is an interesting story that, like life, involved *Direction, Determination, Discouragement, and Discovery*.

## Introduction

**Figure 1:**
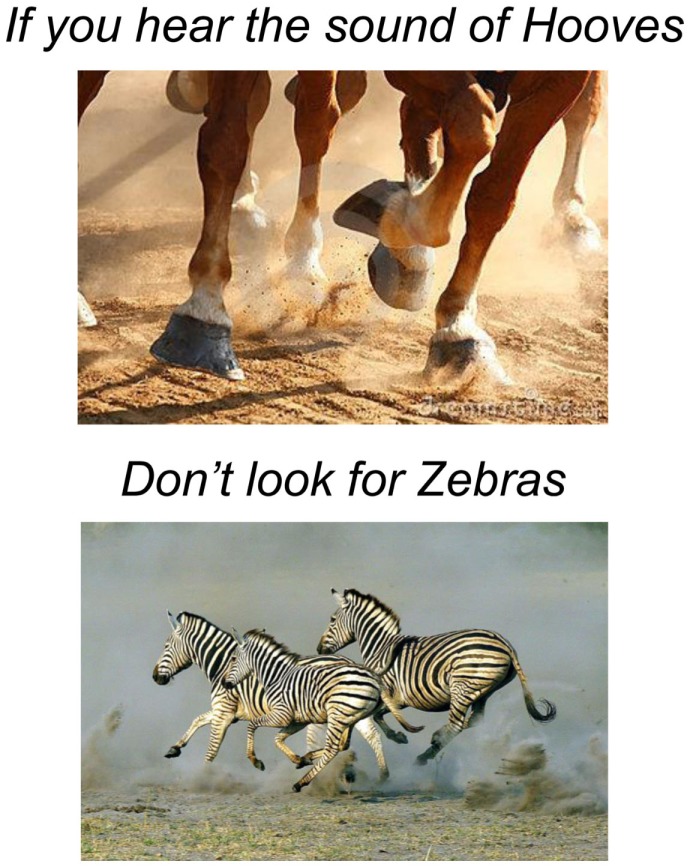
**It is useful sometimes to recognize the obvious rather than look for more complicated explanations in science, or in life**.

A revolution in immunology is underway. Macrophages and innate immunity are now known to be the primary host defense in all animals (1). It had long been thought that adaptive responses (T and B cells) direct innate immunity (2–6). Immunology had it backward. Why? I am reminded of the humorous phrase: “If you hear the sound of hooves, don’t look for zebras.” That is, look for the obvious (Figure 1). Immunology overlooked animal anatomies. Macrophages were the first “immune” cells to appear in evolution, are present in virtually all tissues, and far outnumber other leukocytes (7–9). Despite these anatomical signposts, most immunologists (from the time of Jenner in the 1700s) have been on a quest for the holy grail of immunology: specificity. One cannot blame them. Specific vaccines have resulted in the elimination of world disease scourges such as smallpox and polio.

Meanwhile, macrophages were mainly viewed as “trash disposal units” serving at the bequest of the T and B cells and hidden “under the sink” (10). One might say an “Adaptive Dictator” was in charge (9).

Macrophages were also an enigma. They displayed the stupefying polar-opposite abilities to inhibit proliferation (e.g., kill pathogens) or to promote proliferation (e.g., repair wounds). How could this be? The kill and repair paradox turned out to be based on the elegantly simple and fascinating ability of macrophages to metabolize arginine to either nitric oxide (NO) or ornithine, respectively (11–17). As important as this discovery was, macrophages held another big secret: one that would fundamentally change our understanding of how immune responses occur.

Macrophages’ unique abilities to kill or repair were found in sterile inflammation, where there were no pathogens (foreign antigens), and also in mice without T (or B) cells (14, 16). These observations helped overturn the long-held belief that adaptive responses were necessary to “activate” or “alternatively activate” macrophages (3, 18–20). The importance and independence of innate immunity are highlighted by the oft-overlooked fact that >95% of animals do not have T cells and survive happily in a sea of pathogens, earthworms being an example (7, 8). How? Macrophages! They can kill pathogens within hours. Rapid killing of pathogens is necessary. One bacterium can become the mass of a human in about 4 days, while a T (or B) cell can only become about 16 cells in 4 days. Thus, mathematical considerations alone indicate that clonal proliferation of lymphocytes cannot serve as the primary host defense; this is the job of macrophages throughout the animal kingdom (1). Moreover, in higher animals (e.g., vertebrates) that do have T cells, kill or repair type macrophages (or dendritic cells^1^) necessarily direct T cells to make Th1 or Th2-type responses, respectively (16, 21–23).

Together, these and other results about macrophages have caused a fundamental change in our understanding about how immune systems operate. Macrophages/innate immunity initiates and directs virtually all immune responses, including T and B cells/adaptive immunity (1, 9). Hence, I specifically renamed macrophages *M1* and *M2* to highlight that they, not T cells, are the core of immune systems (16). Of course, once given innate direction by macrophages, the different types of Th1 or Th2-type responses that result can further elevate (or inhibit) M1- or M2-type macrophage responses (1). The macrophage “revolution” did not happen overnight, and is continuing. But how macrophages came out from “under the sink” to occupy the epicenter of immunology is an interesting story that resembles life itself: one of *Direction*, *Determination*, *Discouragement*, and finally *Discovery*. It is about the horses, not zebras, of immunology.

## Setting a Course of Study: Cancer and Immunology

My path to the study of macrophages took awhile. When I entered graduate school in 1974 at the University of Chicago, immunology was pretty new. There was only one immunology course available and few textbooks; so, learning came mainly from reading journals such as the *Journal of Immunology* or *Journal of Experimental Medicine*. I came to believe that the next great immunologic triumph would be more specific vaccines. Having drawn blood in a hospital, as an undergraduate at Syracuse University, patients dying of cancer made a profound impression on me. So, immunology and cancer became my focus: my *Direction*. At this time, cancers appeared to be “foreign” like pathogens (24–26) and viruses were also implicated (27, 28). But, the antigens on cancer cells were weak; they did not readily elicit specific T (or B) cell responses (29).

**Figure 2:**
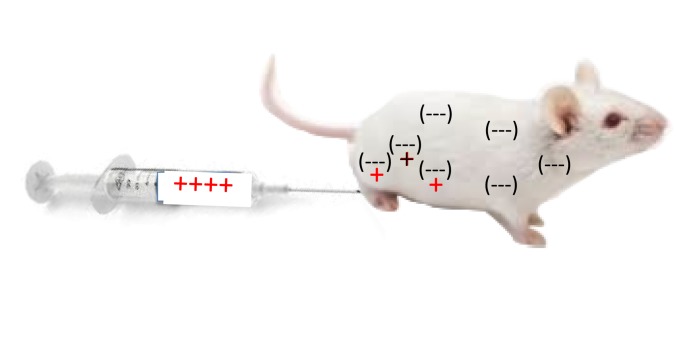
**Injection of bovine serum albumin, chemically modified to be “positively charged,” into a mouse caused it to be retained longer at the site of injection, and stimulated a stronger T cell mediated immune response**.

Having decided I wished to study immunology, I joined Robert Hunter’s lab at the University of Chicago because he was investigating why some antigens were more immunogenic (elicit an immune response) than others in hopes of augmenting anti-cancer and other immune responses (30). While thinking about a Ph.D. project, I realized that animal bodies, as a whole, are “negatively charged” (proteins, sugars, cells, etc.). For example, the electrophoretic separation of most proteins is possible because they migrate from the anode (-) to the cathode (+) at different rates. Cells and other molecules must repel, not stick together, in order to move, to flow. Therefore, I proposed that if a protein antigen was modified to be “positively charged,” it would “stick” in the body longer and elicit a stronger immune response as illustrated in Figure 2. It worked (31).

However, not a lot of people were interested in what made antigens immunogenic in the 1970s (recall the Adaptive Dictatorship), and my manuscript to the *Journal of Immunology* was rejected: a lesson in *Discouragement*. Along the way, I learned that humor is a useful way to deal with *Discouragement*. The south side of Chicago was more ethnically “diverse” than where I had lived. The black friends I developed there had the best sense of humor of any group I have encountered. They used humor artfully to diffuse the increased societal *Discouragement* they typically faced compared to white boys like me.

Investigating how the “charge” of an antigen affects its immunogenicity may seem far removed from the title of this paper. However, studying the biochemistry of antigens and how the immune system handles them provided me with important tools that would help later in figuring out how immune systems operate.

With continued excitement that cancer was “foreign” and with training in what makes antigens immunogenic (particularly *in vivo*), I continued in my *Determination* to boost anti-cancer responses. I joined Bob North’s lab as a postdoc at the Trudeau Institute. Back at Chicago, I had become interested in cytolytic T lymphocytes (CTL) mainly because of Zinkernagel and Doherty’s work, and because Frank Fitch’s lab next door was measuring them (32, 33). Bob, Earl Dye, and I found out that we could use adjuvants (e.g., *C. parvum* or LPS) to augment tumor-specific CTL responses *in vivo* that handily caused tumor rejection (34, 35). This was exciting news. The NIH took notice and began clinical cancer trials trying to boost “killer” lymphocytes (36).

However, a major crack in the “cancer vaccine” armor was becoming apparent to me. It had been reported that mice deficient in T cells did not have an increased incidence of cancer (37). It had also been recently proposed that the immune system could stimulate cancer growth (38). Too, the ongoing NIH clinical cancer immunotherapy trials themselves needed therapy: they did not work (39). The T cell-mediated “immunosurveillance” theory of cancer thus seemed wrong (40): another potential *Discouragement*. However, I was lucky to be at the Trudeau Institute because the studies there mostly focused on understanding diseases processes *in vivo*: an approach I would continue to use. In addition, macrophage “activation” had been discovered there (18, 41) that opened my eyes to another cellular element of the immune system. I also found most interesting the recent observations that macrophages were required for T cells to be activated (42, 43). My postdoctoral studies thus added breadth to my immunologic knowledge that would soon become an advantage: as Pasteur said, “Chance favors the prepared mind.”

## Exploring Macrophages and Solving Their Enigmatic Kill or Repair Abilities

Because of increasing doubts about the “foreignness” of cancer, my introduction to macrophages (and moving to Brown University), I adjusted my *Direction* to focus on the “trash disposal units” of the immune system. I was also going to learn that collaborating with people whose expertise is very different than one’s own can be important. I have come to call it “cross-fertilization.” I teamed up with surgeons Michael Caldwell and Jorge Albina (and Jeff Shearer) who studied wound metabolism, far different from my expertise in immunology. We found macrophages to be the majority leukocytes in sterile wounds, and that they produced the growth/repair-promoting molecule, ornithine (a precursor of polyamines and collagen), that aids in healing (14). But as I previously mentioned, I had learned from studies at the Trudeau Institute that macrophage “activation” was necessary to kill bacterial pathogens (18).

How could one cell perform the polar-opposite activities of growth inhibition (killing pathogens) and growth promotion (healing wounds)? This was vexing indeed. Solving this paradox would eventually lead to the discovery of M1/kill and M2/repair-type macrophages. Not yet, however, as there was still work to be done: *Determination*.

Pursuing wound healing further, we found that macrophages produced so much ornithine in wounds that they markedly and specifically depleted the substrate, arginine, *in vivo*. Could low arginine concentrations in inflammation be important? As I mentioned, I focused on studying immune responses *in vivo*. However, dissecting cellular physiology and functions is sometimes better studied *in vitro*. Having some skills in biochemistry and contemporary tissue culture techniques, I was able to test the hypothesis that low arginine concentrations negatively impact leukocyte functions. Since macrophages were the predominant leukocytes in sterile wounds, we harvested some resident rat peritoneal macrophages and cultured them in varying concentrations of arginine. Opposite from our hypothesis, the more arginine we added to macrophages, the more their functions declined after a few days. We shelved these experiments, thinking we were dealing with some undecipherable *in vitro* artifact. Whereas, this seemed another potential *Discouragement*, I got “lucky.”

While perusing the current *Journal of Immunology* in 1987, I came upon an article by John Hibbs and colleagues reporting that macrophages kill tumor cells using arginine: and only arginine (12).

*Wow* (I will use *Wow* throughout to highlight those rare and wonderful “realization” moments).

**Figure 3:**
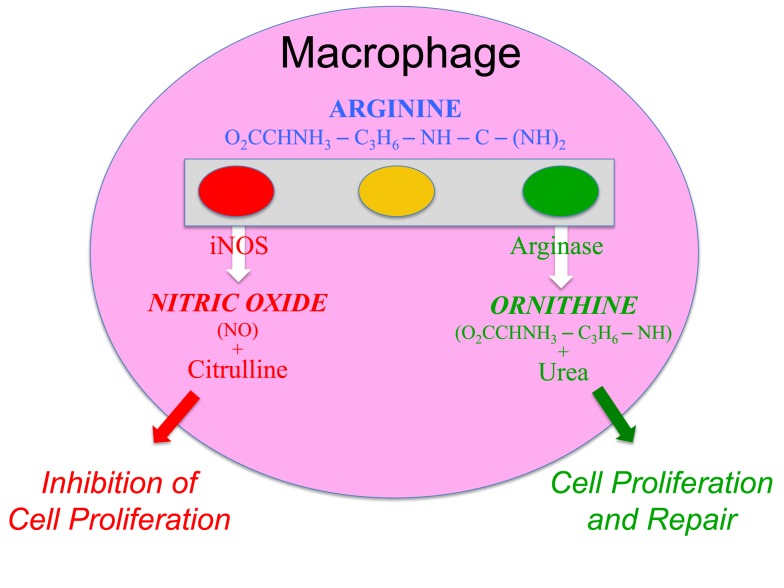
**Macrophages have both iNOS and arginase enzymes that can convert arginine to NO or ornithine, respectively**. Products of each reaction inhibit the opposing reaction, promoting preferential NO or ornithine production.

I realized that the reason our experiments of adding arginine to macrophages decreased (not increased) their functions was that we were unknowingly adding the “fuel” macrophages use to kill, and that the mysterious arginine-derived molecule also killed the macrophages (13). Within months, the arginine-derived killer molecule would be determined to be NO (44). It was a gas (both literally and figuratively humor intended), because now there was an answer to the enigmatic ability of macrophage to kill or repair. Macrophages have the unique ability to metabolize arginine to either make a “Stop” signal or a “Go” signal, as illustrated by the traffic light in Figure 3 [(13), reviewed in Ref. (9, 17)].

## Macrophage Kill and Repair Activities in Wounds and Tumors

**Figure 4:**
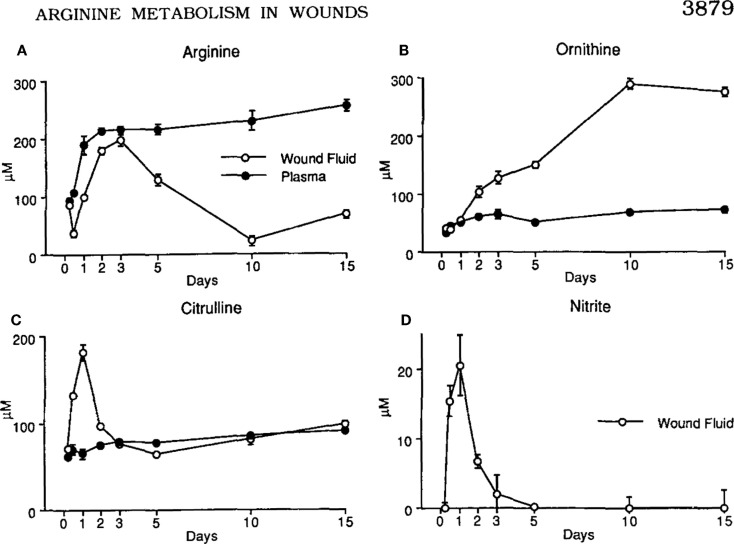
**(A, C)** Following wounding, there is a 1-2 day “burst” of killer NO (measured as Citrulline and NO_2_) *in vivo*, followed by **(B, D)** macrophages metabolizing arginine to the growth-promoting repair molecule, ornithine (and urea), as healing proceeds. From Ref. (14). Copyright 1990. The American Association.

The discovery that macrophages could make either a Stop signal (NO) or a Go signal (ornithine) from arginine was amazing to me. But, were these polar-opposite activities physiologically important? We immediately set about determining if and when macrophages made these Stop or Go molecules *in vivo*. Recall that we already knew that macrophages in healing wounds were making the growth-promoting molecule ornithine. So, we examined if macrophages were also making NO in wounds. They did, but only for a few days after wounding (to kill pathogens if introduced) as shown in Figure 4 (14). I was now convinced that these dual arginine-based kill or repair pathways in macrophages were important *in vivo*.

**Figure 5:**
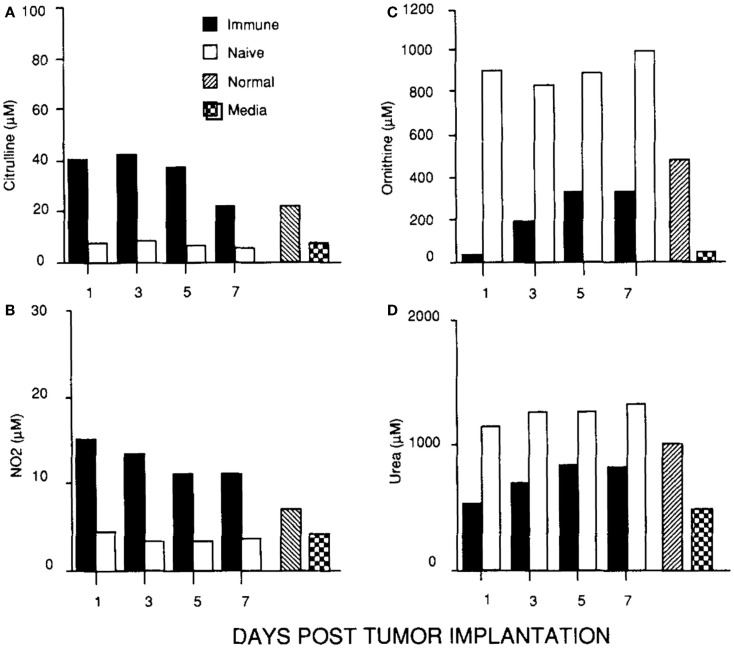
**(C, D)** Macrophages in a growing tumor (naïve) make growth-promoting ornithine (and urea). **(A, B)** Macrophages in a rejecting tumor (immune) make killer NO (and citrulline). From Ref. (45). Copyright 1992. The American Association of Immunologists, Inc.

In parallel with studying macrophages in wounds, I was continuing my cancer studies using an intraperitoneal tumor model. This site allowed me to look at the cellular and molecular events going on inside growing tumors, or in tumors being rejected. I found that macrophages inside growing tumors primarily made ornithine, just like macrophages in healing wounds. Notably, macrophages in growing tumors only made ornithine; there was no initial “burst” of NO as observed following wounding. In marked contrast, macrophages inside rejecting tumors (preimmunized mice) made a lot of NO (and there was a strong intratumor CTL response and IFN-γ production) (45). Thus, macrophages inside growing tumors make a molecule (ornithine) that promotes proliferation, and macrophages inside rejecting tumors make a molecule (NO) that inhibits proliferation as shown in Figure 5.

*Wow* These seminal results in 1990 and 1992 convinced me that macrophage arginine-based repair or kill responses were not only important *in vivo*, but involved with the growth or rejection of cancer: my original *Direction*.

## Involvement of Macrophage Kill or Repair Activities in Many Diseases

The findings that macrophages make proliferation-promoting ornithine during inflammation where cells are growing (healing wounds or cancer), or make proliferation-inhibiting NO where cells are being killed caused me to re-double my *Determination* to studying these macrophage responses in diseases. My family and I moved to the University of Minnesota where a great new lab complex had been constructed for Mike Caldwell, Jeff Shearer, and me. The breadth of immunologic knowledge I had acquired about macrophages at the Trudeau Institute and collaborations with people whose expertise was different than mine would continue to be fruitful.

Along the way, there were some funding and other difficulties: *Discouragement*. For example, as I did not publish a lot of papers, funding agencies were perennially “reminding” me of this (instead of focusing on citation impact). But my *Direction* and *Determination* remained with macrophages.

Damn the torpedoes, full speed ahead! James Farragut, Civil War, 1864.

Having realized from our earlier studies that when macrophages make the gas NO it non-specifically kills everything nearby, I began to wonder if macrophage kill/NO or repair/ornithine responses were involved in other disease processes. For example, it had been observed during several chronic infections that macrophages inhibit specific T (or B) cell responses through “suppressor” activity (46, 47). Knowing this, and having the tools to enhance or inhibit NO production, we were able to show that macrophage “suppressor” activity (measured *in vitro*) could largely be attributed to their production of NO (48). It was also revealed that the presence of red blood cells blocked the NO-mediated suppressor activity (NO binds avidly to hemoglobin) (17). But, there are myriad differences between *in vitro* leukocyte reactions and how the immune system operates *in vivo*, as I have recently discussed (49). I knew that in rejecting tumors (mentioned earlier, Figure 5) that there were both specific CTL and macrophages making a lot of NO (45). This model system allowed me to test if macrophage NO also inhibited T cells *in vivo*. We implanted Alzet Pumps containing *N*-g-monomethyl-l-arginine (iNOS/NO inhibitor) inside rejecting tumors. Doing so elevated the tumor-specific CTL response (50). Thus, NO was thus not simply beneficial against tumors (or pathogens), but also immunoregulatory. If overproduced, NO could inhibit beneficial immune responses *in vivo* [reviewed in Ref. (17)]. In a related connection, we knew from our earlier studies in wounds that tissue disruption causes a short “burst” of NO production as shown in Figure 4 (14). It is now clear that this is an evolutionarily old response that most animals have which serves to “sterilize” the area (in case pathogens are introduced) – something I have termed the “Damage Danger” response (9). It happened upon a surgery resident at the University of Minnesota who was working with the noted transplant surgeon David Sutherland. They were trying to figure out how to improve “islet” transplantation (groups of insulin-producing β cells from the pancreas) for diabetes treatment. As in a wound, we found that injecting islets also caused a short local burst of NO. We were able to show that inhibiting this rapid NO response increased the efficiency of cellular transplantation (51). In another study, we found that β-cell destruction in autoimmune diabetes was also associated with overproduction of macrophage NO and was regulated by insulin (52).

The aforementioned results greatly expanded the “universe” of macrophage NO *in vivo* from that of a host protective molecule to an immunoregulatory molecule and a non-specific tissue-damaging element if overproduced. Subsequent studies have verified the powerful two-edged sword nature of macrophage NO (and ornithine) in many infectious and autoimmune diseases (9, 17, 53–57), as we had originally observed in wounds and tumors (14, 45). Of particular note, overproduction of macrophage NO appears to be causative in atherosclerosis (58–60). Thus, the balance between the macrophage killer (NO) and repair (ornithine) responses now seemed important in both of the two major health problems of modern man: cancer and atherosclerosis.

*Wow* Stay tuned; it gets even better.

## The Road to M1 and M2 Macrophages

### T cells determine immunity: Or do they?

While I was busy studying macrophages, most immunologists continued to view “immunity” in humans (higher animals) from a T cell/adaptive immunity perspective. For example, it had been shown that different strains of mice vary tremendously in their susceptibility to infectious agents. In particular, C57Bl/6 mice were much more resistant to *Leishmania* than were Balb/c mice (61). The difference in resistance correlated with the ability of C57Bl/6 mouse T cells to produce a lot of IFN-γ during infection that activates macrophages to kill the parasite [by now NO was known to be important in killing intracellular pathogens (62)]. In contrast, Balb/c T cells made more IL-4 that did not stimulate NO production, but instead stimulated antibody production, which was ineffective against the parasite. The IFN-γ dominant T cell response came to be known as Th1, while the IL-4 dominant response was called Th2 (2). That hosts mounted very different T cell responses to *Leishmania* was an exciting development because it seemed to explain differences in disease susceptibilities.

But my immunology experiences had taught me that *correlation* is not *causation*. Recall which leukocytes are the most abundant in animals – macrophages – not T cells. The saying that, “If you hear the sound of hooves, don’t look for zebras” was about to take on an important new meaning.

### Macrophage responses vary between individuals independent of T cells

**Figure 6:**
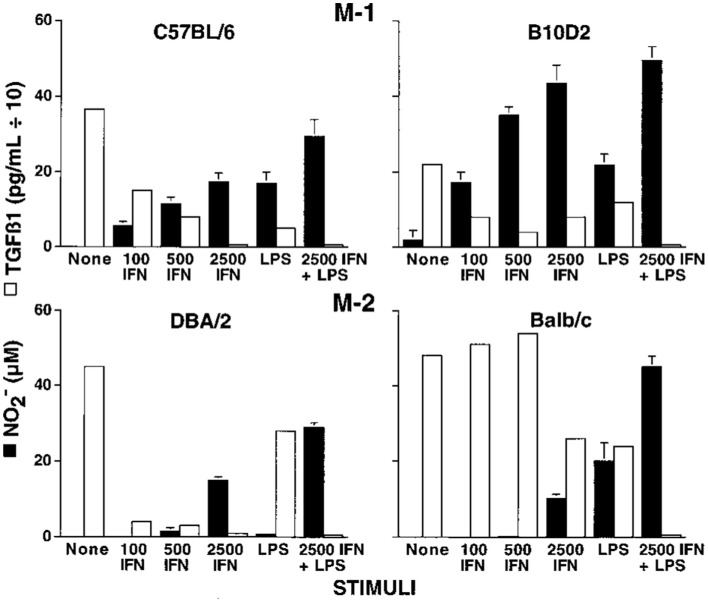
**Dominant NO production by C57B/6 macrophages compared to Balb/c macrophages**. Also, NO production is inversely proportional to macrophage TGF-β production. From Ref. (16). Copyright 2000. The American Association of Immunologists, Inc.

Knowing there were major differences in the T cell responses of different mouse strains to *Leishmania*, I wondered if the macrophage killer and repair responses I was studying also varied. We harvested resident tissue macrophages from C57Bl/6 and Balb/c mice (and a few other strains), and compared their abilities to make the killer molecule NO or the repair molecule ornithine. Note: unlike most, I used resident macrophages, not “elicited” macrophages. Though the cell yield was much lower (more mice needed), it allowed me to look at “resting” macrophages. Resident C57Bl/6 macrophages were much easier to stimulate to make NO (with IFN-γ or LPS) than were Balb/c macrophages. Furthermore, LPS stimulated NO production by C57Bl/6 macrophages, but instead caused increased ornithine production by Balb/c macrophages (16). Thus, using the same stimulus C57Bl/6 mouse macrophages could produce a growth-inhibiting molecule while Balb/c made a growth-promoting molecule. This was very interesting. Also, because the stimuli used were not specific to *Leishmania* the results suggested that differences in macrophage responses between mouse strains were general phenomena. Having an amino acid analyzer available (because of our interest in metabolism), importantly made direct measurement of the arginine-derived kill (NO) and repair (ornithine) molecules possible: a point I will discuss later. We made our own serum-free culture media for these experiments because it was known that serum contains high levels of TGF-β (mainly from lysed platelets) that strongly inhibits macrophage NO production (17). Serum-free media also allowed us to show that macrophages make TGF-β, and when they are stimulated to make NO, TGF-β production goes down, as shown in Figure 6. Subsequent studies have confirmed that TGF-β is a key cytokine that regulates the balance between macrophage NO and ornithine production (1, 16, 17, 63–66).

The differences observed in the responsiveness of C57Bl/6 and Balb/c macrophages to LPS or IFN-γ suggested that resistance to *Leishmania* might involve macrophages. To rule out the influence of T (or B) cells, I compared the ability of C57Bl/6 or Balb/c Nude or SCID macrophages to make NO or ornithine. The results were breathtaking. C57Bl/6 Nude or SCID macrophages made a lot of NO while Balb/c Nude or SCID macrophages did not, just like their normal counterparts (16).

Major *Wow*

The propensity of macrophages to make killer or repair responses was independent of T (or B) cells. Could this also mean that differences in macrophages between individuals (not T cells) determine susceptibility to *Leishmania* or other diseases?

### The discovery and the importance of M1 and M2 macrophages

**Figure 7:**
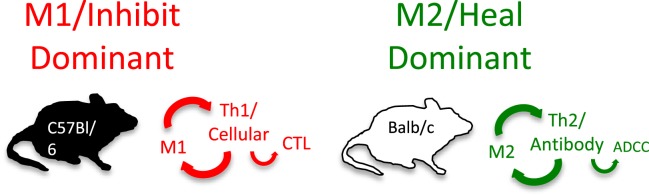
**Macrophages from C57Bl/6 mice make M1-dominant (NO) responses while Balb/c are M2-dominant (ornithine)**. M1- or M2-dominant responses stimulate Th1- or Th2-type responses that can further amplify cellular/CTL and M1, or antibody-type and M2 responses. From Ref. (1) with permission from S. Karger AG, Basel.

As part of investigating macrophage kill or repair responses in different mouse strains, I also wondered whether the propensity of C57Bl/6 or Balb/c T cells to make IFN-γ (Th1) or IL-4 (Th2), respectively, was only true in *Leishmania* infection. It was not. When I stimulated C57Bl/6 or Balb/c spleen cells with Con A (polyclonal stimuli), they made more IFN-γ or IL-4, respectively. Thus, C57Bl/6 and Balb/c mice had a general propensity to make Th1- or Th2-type cytokines. But why? To answer this question, I designed an experiment that perhaps only an immunologist/immunogeneticist could enjoy. We harvested C57Bl/6 × Balb/c F1 spleen cells and depleted the macrophages and red blood cells. Then, we mixed the F1 lymphocytes with macrophages from SCID C57Bl/6 or SCID Balb/c mice and added Con A. C57Bl/6 SCID macrophages caused the T cells to make a Th1-type response (IFN-γ) and Balb/c SCID macrophages caused the same type of T cells to make a Th2-type response (TGF-β) (16). Note: these experiments were possible because F1 T cells do not recognize either parent as foreign. Differences in macrophage responses alone could explain the ability of different mice to mount Th1- or Th2-type responses and in turn their susceptibility to diseases. Macrophages direct T cells as illustrated in Figure 7.

Discovery *Wow*

**Figure 8:**
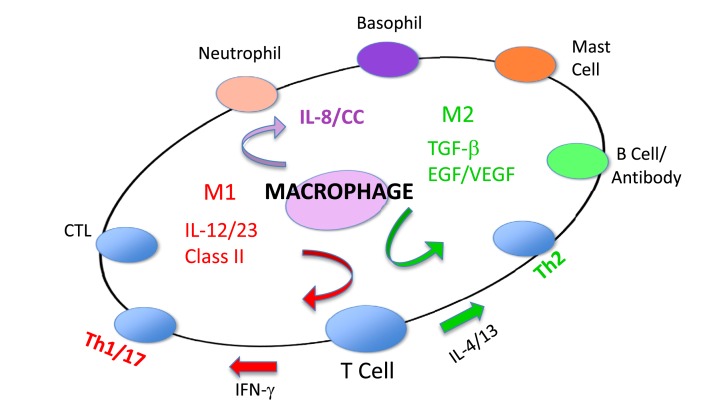
**Macrophages initiate and direct other immune responses**. For example, M1-type responses (e.g., through IL-12 and antigen presentation) direct T cells to become cytolytic T cells and produce IFN-γ that further elevates M1 activity. In contrast, M2-type macrophages cause T cells to produce molecules like IL-4 and TGF-β that cause B cells to produce antibody and elevate M2 responses. From Ref. (1) with permission from S. Karger AG, Basel.

Because of their polar-opposite kill and repair activities, the independence of these responses from T cells, and that these types of responses stimulated Th1- or Th2-type responses, I renamed macrophages *M1* and *M2* to highlight the importance of innate immunity over adaptive immunity (16). M1/inhibit and M2/heal responses and their impacts on inflammation and immunity are illustrated in Figure 8. The long-held belief that “zebras” (T cells) were necessary to “activate” or “alternatively activate” macrophages was incorrect and even backward (3–6, 19, 20). The adaptive dictator had been overthrown. The horses/macrophages were the center of the immune “solar” system. Anatomy was proven correct after all.

## M1 and M2 Macrophage Responses Defined

### Causative functions and molecules that affect health

**Figure 9:**
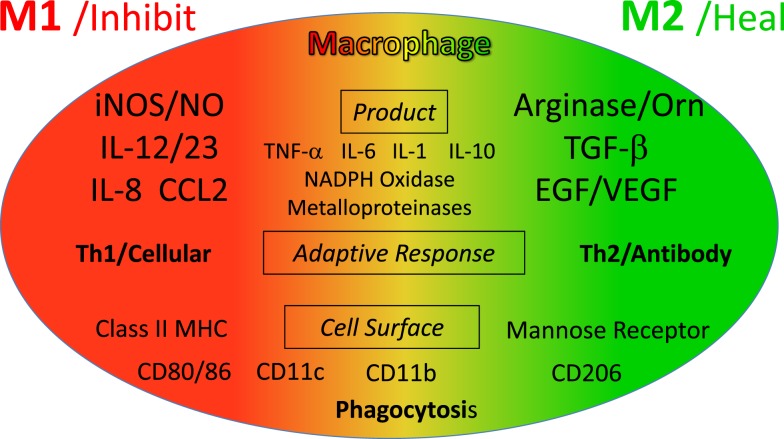
**Cytokines and other molecules associated with M1/inhibit or M2/heal-type responses**. Certain products (middle) have been associated with both M1 and M2-type responses and can be thought of as general “inflammatory” cytokines or factors. From Ref. (1) with permission from S. Karger AG, Basel.

As described, M1 and M2 macrophage responses were originally defined *in vivo* by the preferential production of the *causative* functional molecules NO or ornithine which inhibit or promote proliferation. Since then M1 or M2 macrophages responses have been shown to occur in concert with certain other molecules that can aid in characterization. As shown in Figure 9, M1 responses are linked with IL-12 and IL-8/CCL production, and cell surface expression of CD 80 or 86 that attract or killer cells like neutrophils and/or stimulate Th1 responses such as CTL and further M1-type activation. M2 responses are associated with TGF-β, and growth factor production (e.g., VEGF or EGF), cell surface expression of CD163 or 206, and the propensity to stimulate Th2 responses such as antibody production and further amplification of M2-type responses, as illustrated in Figure 7. Macrophages also make TNF-α, IL-6, IL-1, IL-10, NADPH oxidases, and metalloproteinases. However, these molecules are produced by many macrophage populations and are not as clearly diagnostic of M1 or M2-type responses as NO or ornithine or the other molecules listed in Figure 9.

### Other markers of macrophages

As mentioned above, in addition to molecules that are closely linked to M1 and M2-type macrophage responses, macrophages produce a variety of other what can be called “inflammatory” molecules. However, as mentioned earlier with T cells and *Leishmania* resistance, *correlation* is not *causation*. In this regard, some refer to M1 or M2-type responses as “pro-inflammatory” or “anti-inflammatory.” But, this practice is misleading. For example, M2-type responses dominate in wounds as shown in Figure 4. As anyone knows a wound is hardly “anti-inflammatory.” Wherever macrophages accumulate, there is inflammation. So, molecules like IL-1 or IL-6 are more diagnostic of the presence of macrophages rather than of M1 or M2-type responses. In turn, the use of these inflammation-type markers by some laboratories has lead to classifying macrophage populations as M1 or M2-type that are not. In a related vein, techniques like transcriptomics and FACS (67–69) are creating ever-enlarging lists of other “markers” being used in analysis of macrophage populations, and individual laboratories often use their own particular markers. Not surprisingly then, these variations in the “metrics” used has created confusion in trying to classify macrophage populations. For example, various different names have been proposed for macrophages such as: M2 a, b, c; type II; or regulatory macrophages (69–71). But, such “subsets” do not have distinct functions associated with them *in vivo* like M1/kill or M2/repair. To try and address this confusion, a new “nomenclature” was recently suggested to classify macrophages (72). However, the nomenclature suggested is also not based on functions, but mainly on what cytokine or factor was added to macrophages *in vitro*. In this connection, the various combinations of different cytokines, agonists, or markers that can be employed in stimulating or analyzing macrophages are very high. But the number of macrophage functions is small.

**Figure 10:**
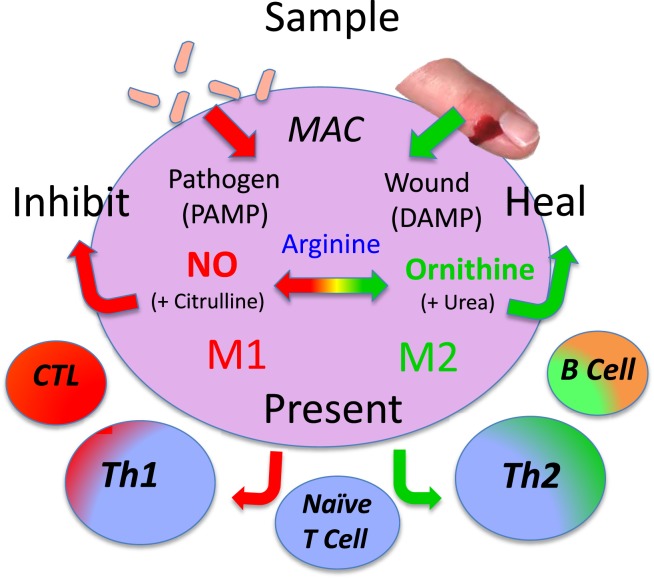
**Macrophages have four basic SHIP functions [sample, heal, inhibit and present (antigen)] that allow them to recognize pathogens or injury, and respond directly (or indirectly by presenting antigens) to engender responses that provide optimal host protection**. From Ref. (1) with permission from S. Karger AG, Basel.

Specifically, macrophages have four core functions called SHIP [sample, heal (M2), inhibit (M1), and present (antigen)] as shown in Figure 10. Therefore, to best understand macrophage populations it is important to stay focused on analyzing them by functions, such as SHIP, that are known to affect health as recently discussed (1, 49).

### Heterogeneity and plasticity are not macrophage functions

Infections, cancer, or other inflammatory conditions are ever evolving as disease protection or progression occurs. This fact and that macrophages have the unique ability to drastically change their physiology to protect hosts necessarily means that macrophage populations are heterogeneous. Plasticity, a term I coined in 2001 (17), was later popularized by my now deceased friend, Bob Stout, and his wife Jill Suttles (73). Plasticity is a useful word to describe the unique ability of macrophages to change their functions. Beyond this, some have posited that macrophages are like a “color wheel” (74). But, it is important to note that heterogeneity, plasticity, or color wheels are not functions that affect health. For example, as we saw, M2-type macrophages inside tumors promote tumor growth while M1-type inhibits tumor growth as illustrated in Figure 5 (45). These findings have since been verified in many human cancers (75–78). Therefore, if one has cancer, one would wish to decrease intratumor M2-type and increase M1-type macrophages. Heterogeneity, plasticity, or color wheels will not stop cancer. So, though such terms are useful in describing the fungibility of macrophage populations, again, advancing health will only come from knowing what macrophage functions are by measuring them, so they can be modulated as needed (49).

## Parallel Elements of the “4Ds” in Scientific Investigations and Life

As I said at the outset, events in biomedical research can resemble life itself: there is *Direction, Determination, Discouragement, and Discovery*.

I feel most fortunate in having an upbringing that allowed me to become a scientist. I have met many people along the way with towering intelligence, but who did not have such an advantage and who work at difficult manual labor jobs. I was also fortunate in being influenced early in my *Direction* by seeing the devastation that cancer can bring, and in picking immunology to study cancer. Regarding *Determination*, many people work hard and I am not unique. But in science, one should not “fall in love” with one’s ideas. As described here, *Wow*-type moments often come through serendipity: when one must trust the results and abandon existing hypotheses. Recognizing that cancer was not overtly “foreign”, and focusing on macrophages/innate immunity was one of those moments for me. One cannot know everything about an immense field such as immunology. However, I think cultivating a breadth of knowledge helped prepare me for times when, “Chance favors the prepared mind”, such as elucidating the arginine-based dual M1/inhibit or M2/heal functions of macrophages (16, 17). As I mentioned earlier, my belief in the importance of macrophages was bolstered by the findings that they were necessary stimulators of T cells (21, 22). Also, toll receptors were identified on macrophages in the 1990s (79–81) that provided additional support for my concept that macrophage responses are independent of T cells, and also initiate immune responses. As a sidebar here, I worked with Ralph Steinman some when I was a postdoc at the Trudeau Institute in the early 1980s and enjoyed his company. He is credited with discovering dendritic cells (21). It may well be from me having a lack of folds in my cerebrum, but I have always found it simpler to consider dendritic cells as a subset of macrophages (17). So, if you are dendritic cell “fan,” you could substitute those words for macrophages in some places in this treatise. But, it does not change the larger point that M1/M2-type macrophages have the unique ability to display polar opposite kill or repair responses and that innate immunity directs adaptive immunity. I will leave the macrophage versus dendritic cell discussion to others (22, 23).

As I said earlier, there was *Discouragement* along the way. As anyone in biomedical research knows, funding one’s work can be difficult and frustrating. In my own case, I knew deep down I had found something beautiful about the immune system in the *Discovery* of M1/inhibit and M2/heal-type macrophages in 2000. But dogma can be slow to change, and I did not get an important NIH grant renewed. Having had my own lab for many years, I did not wish to work for someone else, and I left the University of Minnesota to do other things for a while. One very satisfying thing I did was coach my sons and daughter’s basketball teams. I feel sports teach important life lessons, such as fair play, and being gracious in victory or defeat.

In the mid 2000s, my M1/M2 *Discovery* started to be appreciated. Of course, medical research is very competitive; so, it was not surprising that some tried to rewrite history about macrophage subsets (69, 70). In particular, it is “curious” that some reviews about M1 or M2-type tumor-associated macrophages (82, 83) somehow overlook the seminal studies that elucidated the M1 growth-inhibiting and M2 growth-promoting macrophages in tumors and wounds [e.g., Figures 4 and 5; (14, 45)]. Like I mentioned about sports and fair play, it is appropriate to try and acknowledge other’s studies. In this connection, Zouhair Atassi recognized that I was the origin of the M1/M2 macrophage concept, and asked me to write a review for *Critical Reviews in Immunology* in 2012 that I entitled, “M1and M2 Macrophages: Oracles of Health and Disease” (9).

**Figure 11:**
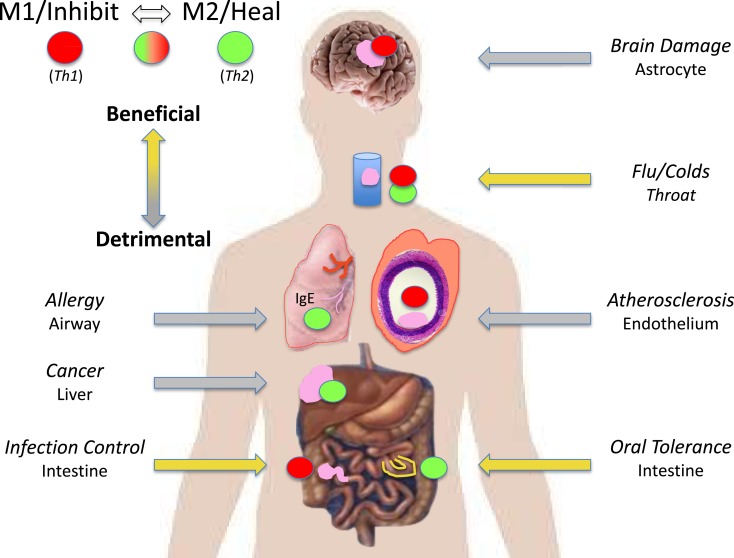
**M1/inhibit or M2/heal-dominant macrophage responses (or mixtures) can each be beneficial or detrimental depending on the disease circumstance**. For example, M1/Th1-dominant responses are required to fight many infections (left). But, M1/Th1 responses are also causative of destructive inflammatory conditions in the brain and in atherosclerosis (right).

The M1/M2 concept has fundamentally changed our understanding of what “immunity” is by showing the biochemical bases for the unique abilities of macrophages to kill or repair, and that macrophages/innate immunity initiate and direct immune responses throughout the animal kingdom, including adaptive immunity in humans (1, 7, 8, 16). M1/M2 has not only stood the test of time, but thousands of publications indicate that interest in macrophages/innate immunity and clinical applications are ever increasing. I apologize for not mentioning many good results about M1 and M2 macrophages here, but readers should be able to track down studies of interest from the reference list. I am particularly gratified that there is great potential for the successful immunotherapy of cancer by modulating M2 into M1-type macrophages (76, 84): my original *Direction*. Indeed, *Science* magazine referred to 2013 as the year of immunotherapy. Some examples of the myriad diseases where the powerful two-edged sword nature of M1 or M2-dominant macrophage responses can be beneficial or detrimental are illustrated in Figure 11. Macrophages are indeed the oracles of health or disease.

I hope you got a “charge” (humor from my Ph.D. earlier) out of “Anatomy of a Discovery” and experience your own *Wows*.

## Conflict of Interest Statement

The author declares that the research was conducted in the absence of any commercial or financial relationships that could be construed as a potential conflict of interest.

## Footnotes

^1^Macrophages and dendritic cells are both derived from “myeloid” precursor cells. Whether dendritic cells are a unique cell type or a specialized type of macrophage is less important than the bigger point that both are innate-type cells that can present antigens to T cells. So, for convenience, “macrophage” will be used throughout here. Readers are directed elsewhere for discussions about macrophages and dendritic cells (21–23).
